# The dynactin subunit DCTN1 controls osteoclastogenesis via the Cdc42/PAK2 pathway

**DOI:** 10.1038/s12276-020-0406-0

**Published:** 2020-03-24

**Authors:** Yong Deok Lee, Bongjun Kim, Suhan Jung, Haemin Kim, Min Kyung Kim, Jun-Oh Kwon, Min-Kyoung Song, Zang Hee Lee, Hong-Hee Kim

**Affiliations:** 1 Department of Cell and Developmental Biology, BK21 Program and DRI, Seoul National University, 101 Daehak-ro, Jongno-gu, Seoul, 03080 Korea; 2 Arthritis and Tissue Degeneration Program, David Z. Rosensweig Genomics Research Center, Hospital for Special Surgery, 535 East 70th Street, New York, 10021 NY USA

**Keywords:** Mechanisms of disease, Experimental models of disease

## Abstract

Osteoclasts (OCs), cells specialized for bone resorption, are generated from monocyte/macrophage precursors by a differentiation process governed by RANKL. Here, we show that DCTN1, a key component of the dynactin complex, plays important roles in OC differentiation. The expression of DCTN1 was upregulated by RANKL. The inhibition of DCTN1 expression by gene knockdown suppressed OC formation, bone resorption, and the induction of NFATc1 and c-Fos, critical transcription factors for osteoclastogenesis. More importantly, the activation of Cdc42 by RANKL was inhibited upon DCTN1 silencing. The forced expression of constitutively active Cdc42 restored the OC differentiation of precursors with DCTN1 deletion. In addition, PAK2 was found to be activated by RANKL and to function downstream of Cdc42. The DCTN1-Cdc42 axis also inhibited apoptosis and caspase-3 activation. Furthermore, the anti-osteoclastogenic effect of DCTN1 knockdown was verified in an animal model of bone erosion. Intriguingly, DCTN1 overexpression was also detrimental to OC differentiation, suggesting that DCTN1 should be regulated at the appropriate level for effective osteoclastogenesis. Collectively, our results reveal that DCTN1 participates in the activation of Cdc42/PAK2 signaling and the inhibition of apoptosis during osteoclastogenesis.

## Bone remodeling: Regulatory protein identified

A critical mechanism for maintaining bone health uncovered by scientists in South Korea could provide insights into bone disease development. Bone remodeling is a lifetime process of bone generation that ensures bones remain healthy. Osteoclasts (OC), cells that break down bone, differentiate from white blood cell populations. Disruption to OC formation and function plays a critical role in bone diseases, yet the regulatory mechanisms in OC generation are unclear. Hong-Hee Kim at Seoul National University and co-workers investigated the role of a protein called DCTN1, which is involved in skeletal assembly processes. The team found that inhibiting DCTN1 suppressed the expression of key proteins needed for OC formation in cell cultures and mouse models. Overexpressing DCTN1 was equally damaging, suggesting the protein plays a key regulatory role.

## Introduction

Osteoclasts (OCs) are specialized bone-resorbing cells that play an essential role in physiological bone remodeling. The dysregulation of OC number or function leads to abnormal bone turnover and is associated with various bone diseases^1,2^. Therefore, understanding the mechanism by which osteoclastogenesis is regulated is imperative for the development of new therapeutic strategies for bone diseases. The formation of OCs from monocyte/macrophage lineage precursor cells is triggered by the interaction of the differentiation factor receptor activator of nuclear factor kappa B ligand (RANKL) with its receptor RANK. Upon RANKL binding, tumor necrosis factor receptor-associated factors are recruited to RANK, and multiple intracellular signaling pathways, including mitogen-activated protein kinase (MAPK) signaling cascades and the phosphatidylinositol 3-kinase (PI3K)/Akt pathway, are activated^3,4^. These signaling cascades induce and/or activate several transcription factors that promote the transcription of OC marker genes. Among the transcription factors involved in this process, NFATc1 and c-Fos are essential for the expression of OC genes during differentiation^1,5^.

Dynactin was the first identified activator of the cytoplasmic motor protein dynein, which mediates various cellular processes, including vesicular transport, mitotic spindle formation, and cytokinesis, to be identified^6,7^. Dynactin is a large protein complex comprised of 11 different polypeptide subunits: p150^Glued^ (DCTN1), p50 (DCTN2), p24 (DCTN3), p62 (DCTN4), p25 (DCTN5), p27 (DCTN6), actin-related protein (ARP) 1/11, actin, and actin capping protein (CapZ) α and β^6^. As DCTN1 can simultaneously bind to both microtubules and dynein, it functions as the critical regulator of the processivity of dynein in microtubule-based transport^6,7^.

DCTN1 has also been implicated in cytoskeleton assembly and organization^8^. The small GTPase Cdc42 regulates actin cytoskeletal architecture to allow cell shape changes and migration^9^. A previous study showed that the reorientation of the microtubule-organizing center is mediated by dynein/dynactin in a Cdc42 activation-dependent manner to allow the migration of lysophosphatidic acid-stimulated fibroblasts^10^, indicating cooperation between dynactin/dynein and Cdc42 for cytoskeleton regulation by dynactin. The most well-characterized downstream targets of Cdc42 are the p21-activated kinase (PAK) family of Ser/Thr kinases^11^. The activation of PAKs can lead to the stimulation of MAPKs (ERK1/2, p38, and JNK) in response to various stimuli^12,13^. However, the role of the link between the Cdc42/PAK and MAPK pathways in the function of dynein/dynactin has not been explored.

Through its microtubule-binding properties, DCTN1 may participate in the regulation of intracellular signaling, as the interaction of signaling molecules with microtubules facilitates signal transmission to downstream targets^14^. In NIH3T3 fibroblasts, approximately one-third of all MAPKs have been found to be associated with microtubules^14,15^. DCTN1 has been shown to interact with MKK3/6 in HeLa cells, and DCTN1 knockdown reduces the phosphorylation of MKK3/6 and p38 MAPKs in response to sorbitol^16^. Moreover, the recruitment of Akt to microtubules is mediated by DCTN1 and sustains the phosphorylation of Akt^17^. However, the precise relationships between signaling molecules, dynactin, dynein, and microtubules in the spatial organization and activation of signaling components remain unknown.

In this report, we found that DCTN1 was upregulated by RANKL in an early phase of osteoclastogenesis. Abnormal levels of DCTN1 induced by gene silencing or forced overexpression inhibited the differentiation of OCs both in vitro and in vivo. The regulatory effects of DCTN1 on OCs were mediated by the Cdc42/PAK2 pathway, which modulated the activation of MAPKs and caspase-3 expression. Our study revealed a previously unrecognized role of DCTN1 in the regulation of intracellular signaling and apoptosis during OC differentiation.

## Materials and methods

### Antibodies and reagents

Anti-NFATc1, anti-c-Fos, anti-Cdc42, and anti-DCTN1 were purchased from Santa Cruz Biotechnology (Paso Robles, CA, USA). Anti-phospho-PAK1 was purchased from Cell Signaling Technology (Beverly, MA, USA), anti-phospho-PAK2 was purchased from GeneTex (Irvine, CA, USA), and anti-phospho-PAK4 was purchased from Bioss Antibodies (Woburn, MA, USA). All other antibodies were purchased from Cell Signaling Technology. HiPerFect was purchased from QIAGEN (Hilden, Germany). Human soluble RANKL and M-CSF were purchased from Pepro-Tech (Rocky Hill, NJ, USA). Cell counting kit-8 (CCK) was obtained from Dojindo (Kumamoto, Japan).

### Bone marrow-derived macrophage preparation

The marrow of the tibiae and femurs of 5-week-old female ICR mice was flushed with α-MEM (Welgene, Daegu, Korea). After removal of erythrocytes with hypotonic buffer, the cells were cultured in α-MEM containing 10% fetal bovine serum (FBS), penicillin (100 units/ml), and streptomycin (100 μg/ml) for 24 h. Non-adherent cells were then collected and further cultured on petri dishes in α-MEM containing 10% FBS, penicillin, streptomycin, and M-CSF (30 ng/ml) for 3 days. Unattached cells were removed, and the remaining adherent cells were collected and used as bone marrow-derived macrophage (BMMs).

### OC differentiation and tartrate-resistant acid phosphatase staining

BMMs (3 × 10^4^ cells/well in 48-well plates) were cultured in OC differentiation medium (ODM) consisting of α-MEM containing RANKL (200 ng/ml), M-CSF (30 ng/ml), 10% FBS, penicillin, and streptomycin for 4 days. The culture medium was changed on day 2. The cells were fixed with 3.7% formaldehyde for 20 min at room temperature and permeabilized with 0.1% Triton X-100 for 5 min. After two washes with PBS, the cells were stained for tartrate-resistant acid phosphatase (TRAP) activity using the Leukocyte Acid Phosphatase Assay Kit from Sigma (St. Louis, MO, USA) according to the manufacturer’s instructions. The cells were then washed with distilled water and observed under a light microscope. TRAP-positive cells containing more than three nuclei were considered differentiated OCs. BMMs cultured in ODM for 2 days were considered prefusion OCs (pOCs). For the liquid assay of TRAP activity, 150 µl of TRAP solution [4.93 mg of p-nitrophenyl phosphate (PNPP) in 3.75 ml of 0.5 M acetate solution mixed with 1.25 ml of tartrate acid solution] was added to fixed and permeabilized cells followed by incubation at 37 °C for 30 min. The reaction was then stopped by adding 0.5 M NaOH, and the absorbance was measured at 450 nm using an ELISA reader.

### Gene knockdown

Target gene-specific siRNA oligonucleotides were purchased from Invitrogen (Carlsbad, CA, USA). BMMs were transfected with siRNA oligonucleotides (40 nM) using HiPerFect (QIAGEN) following the manufacturer’s instructions.

### In vitro resorption assay

BMMs were seeded on dentin slices (Immunodiagnostic Systems, Boldon, United Kingdom) in α-MEM containing 10% FBS, penicillin, and streptomycin. The following day, the cells were transfected with siRNA and then cultured in ODM for 9 days. After treatment with 5% sodium hypochlorite for 10 min, the dentin slices were wiped with a cotton swab and rinsed with distilled water to remove the cells. The area and depth of the resorption pits were measured with a Zeiss LSM 5 PASCAL laser-scanning microscope (Carl Zeiss, Oberkochen, Germany).

### Confocal microscopy

BMMs were seeded on glass coverslips in 24-well plates at a density of 5 × 10^4^ cells per well. Cultured cells were fixed with 3.7% formaldehyde and permeabilized with 0.1% Triton X-100. Fixed cells were blocked with 1% BSA in PBS, incubated with primary antibody (1:100) at 4 °C overnight, and washed with PBS. The cells were then incubated with secondary antibody (1:250) for 2 h and counterstained with 4′,6-diamidino-2-phenylindole (DAPI). The cells were observed under a Zeiss LSM 5 PASCAL laser-scanning microscope with a 400 X objective (CApochromat/1.2 WCorr).

### Actin-ring staining

BMMs transfected with siRNA were cultured on 12-mm round cover glasses in 24-well plates with ODM for 4 days. The cells were fixed with 3.7% formaldehyde and permeabilized with 0.1% Triton X-100. The cells were then stained with Alexa Fluor 633- or Alexa Fluor 488-conjugated phalloidin (Invitrogen) for 1 h at room temperature.

### Real-time PCR

Total RNA was isolated using TRIzol reagent (Invitrogen), and cDNA synthesis was performed with 3 μg of total RNA and Superscript II reverse transcriptase (Invitrogen) according to the manufacturer’s instructions. cDNA was amplified with SYBR Green Master Mix reagents (Kapa Biosystems, MA, USA) using an ABI 7500 instrument (Applied Biosystems, Carlsbad, CA, USA). The PCR conditions were 40 cycles of denaturation at 95 °C for 3 sec and amplification at 60 °C for 33 sec. The mRNA expression level was calculated using the 2^−ΔΔCT^ method and normalized to the β-actin level. All primer sets for real-time PCR are listed in Supplementary Table 1.

### Retroviral transduction

pcDNA3-EGFP-Cdc42 Q61L (constitutive active form) and pcDNA3-DCTN1 plasmids were kindly provided by Dr. Stefan Liebau (Department of Neurology, Ulm University, Ulm, Germany). pRetro-XT-PAK2-CFP was purchased from Addgene (Watertown, MA, USA). The coding sequences of Cdc42 Q61L, DCTN1, and PAK2 were each subcloned into the pMX vector. For retroviral packaging, Plat-E cells were transfected with pMX-Cdc42 Q61L, pMX-DCTN1, or pMX-PAK2 and cultured for 2 days. The culture medium containing retroviruses was collected and filtered with a 0.45-μm syringe filter (Satorius, Göttingen, Germany). BMMs were infected with retrovirus-containing culture medium in 10 μg/ml hexadimethrine bromide (polybrene; Sigma) for 24 h.

### Western blotting

Whole cell extracts were prepared by lysing cells with RIPA buffer. Equal amounts of protein were separated on polyacrylamide gels and transferred onto nitrocellulose membranes. After blocking for 1 h with 5% skim milk in Tris-buffered saline containing 0.1% Tween 20, the membranes were incubated overnight at 4 °C with primary antibody. The next day, the membranes were incubated with horseradish peroxidase-conjugated secondary antibody and developed using enhanced chemiluminescence reagents.

### Cell viability assay

BMMs transfected with DCTN1 or control siRNA were cultured in ODM. The cells were incubated with 10% CCK solution in cell culture medium for 1 h at 37 °C. The optical density was then measured with an ELISA reader (iMARK Microplate Absorbance Reader, Bio-Rad, Hercules, CA, USA) at 450 nm.

### Terminal deoxynucleotidyl transferase dUTP nick end labeling (TUNEL) assay

BMMs transfected with control or DCTN1 siRNA were incubated with ODM for 2 days. The TUNEL assay was performed using an In Situ Cell Death Detection Kit (Roche, Basel, Switzerland) according to the manufacturer’s instructions.

### Cdc42 and Rac1 activity assay

BMM cells (1 × 10^6^) were cultured in a 60-mm culture dish and then transfected with control or DCTN1 siRNA. After 24 h, the cells were serum-starved for 4 h and stimulated with RANKL (400 ng/ml) for different time periods. For Cdc42 and Rac1 activity assays, cell lysates were pulled down using the Active Cdc42/Rac1 Assay Kit (Millipore, MA, USA) according to the manufacturer’s instructions. Briefly, the cells were lysed with lysis buffer (125 mM HEPES, pH 7.5, 570 mM NaCl, 5% IPEGAL CA-630, 50 mM MgCl_2_, 5 mM EDTA, and 10% glycerol) from the kit. The cell lysates were incubated with PBD (p21-activated kinase binding domain)-conjugated beads at 4 °C on a rocker. The beads were rinsed three times in lysis buffer. The precipitated active forms of Cdc42 and Rac1 were detected by western blotting.

### Calvarial bone resorption model

ICR mice (5 weeks old) were purchased from OrientBio (Seongnam, Korea). All mice were fed a regular diet and water and maintained on a 12-h light/dark cycle in a specific pathogen-free animal facility of Seoul National University School of Dentistry. All animal experiments were performed with the approval of the Institutional Animal Care and Use Committee at Seoul National University. Control or DCTN1 siRNA (20 µM; 30 µl) was mixed with Lipofectamine 2000 (10 µl; Invitrogen) and injected onto the calvariae of 5-week-old female mice (*n* = 5 per group) three times at 2-day intervals. One day after the first injection, collagen sponges soaked in PBS or RANKL (10 μg) were surgically inserted into the center of the calvariae. The mice were sacrificed on day 7, and the calvariae were collected for TRAP staining and microcomputed tomography (μCT) analysis (SkyScan, Aartselaar, Belgium; 40 Kv, 250 μA, pixel size: 7.61, threshold maximum: 210 and threshold minimum: 100).

### Histology and histomorphometry

Mouse calvariae were fixed in 4% paraformaldehyde and decalcified in 12% EDTA for 4 weeks. The calvariae were then dehydrated in 70 to 100% ethanol and embedded in paraffin. Tissue Section (5 μm thick) were used for TRAP staining. Histomorphometric analysis was performed as described using the Osteomeasure program (OsteoMetrics, Atlanta, GA, USA)^18^.

### Statistical analyses

All in vitro experiments were repeated at least three times. All quantitative experiments were performed at least in triplicate. The data are shown as the mean ± SD. Student’s *t*-test (unpaired, two-tailed) was used to determine the significance between two groups. A *P*-value less than 0.05 was considered significant.

The supplementary information contains more descriptions of the experimental methods.

## Results

### DCTN1 is upregulated by RANKL in an early stage of OC differentiation

**Fig. 1:**
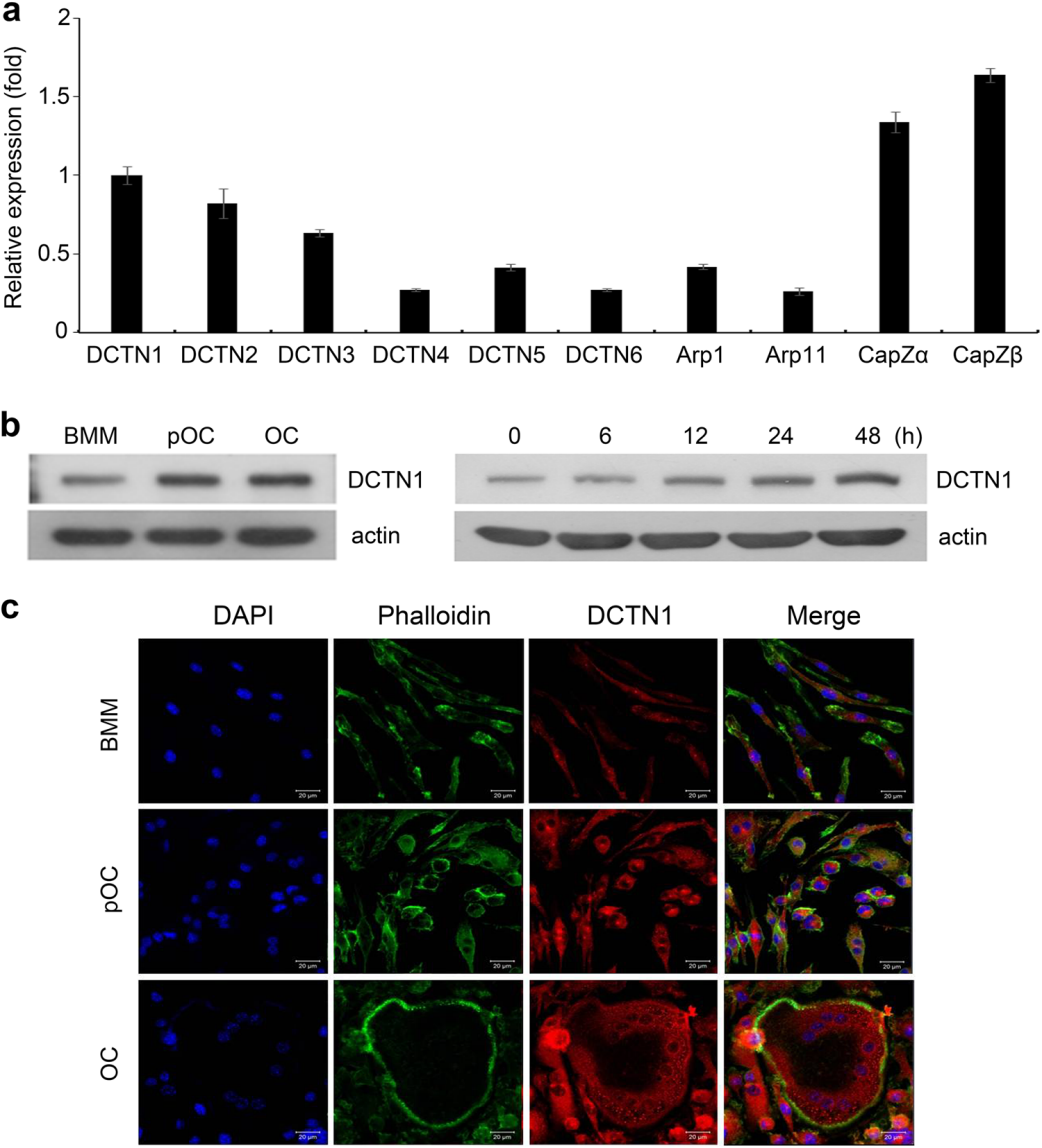
DCTN1 is upregulated during OC differentiation. **a** BMMs were cultured in OC differentiation medium (ODM) containing 200 ng/ml RANKL for 2 days. DCTN1-6, Arp1/11, and CapZα/β mRNA levels were analyzed by real-time PCR. **b** BMMs were cultured in ODM for 2 (pOC) or 4 days (OC) or for the indicated number of hours. DCTN1 protein levels were determined by western blotting. **c** BMMs were cultured in ODM for 2 (pOC) or 4 days (OC). The cells were immunostained using an antibody against DCTN1 and then incubated with a Cy3-conjugated secondary antibody. F-actin was stained with Alexa Fluor 488-conjugated phalloidin.

First, we examined the expression levels of dynactin subunits during osteoclastogenesis. Bone marrow-derived macrophages (BMMs) were cultured for 2 days in osteoclastogenic medium containing M-CSF and RANKL, and mRNA levels were quantitated by real-time PCR. The mRNA levels of CapZα and CapZβ were slightly higher than the mRNA level of DCTN1, while the mRNA levels of other subunits were lower levels than the mRNA level of DCTN1 (Fig. 1a). Since DCTN1 has been suggested to play the most important role in the function of dynactin, we further analyzed DCTN1 expression at different stages of osteoclastogenesis. BMMs were cultured in osteoclastogenic medium and subjected to western blotting. DCTN1 protein levels were elevated in pOCs (cultured with RANKL for 2 days), and this increase in expression was sustained until full differentiation into mature OCs (Fig. 1b). Analysis of BMMs treated with RANKL for 6~48 h revealed that DCTN1 protein expression increased at 12 h and was maintained at this increased level throughout the 48-h treatment period.

Dynactin has been shown to localize to various membrane organelles, including early/late endosomes, phagosomes, mitochondria, the plasma membrane, and vesicles in the Golgi region and in the organelle-free cell periphery in macrophages^19^. Therefore, we next investigated the subcellular localization of DCTN1 during osteoclastogenesis by immunostaining with a DCTN1-specific antibody. DCTN1 was broadly expressed in the cytosol of BMMs and pOCs (Fig. 1c). However, in mature OCs, DCTN1 was restricted to the cell periphery and the area surrounding the nuclear membrane.

### DCTN1 is required for OC differentiation

**Fig. 2:**
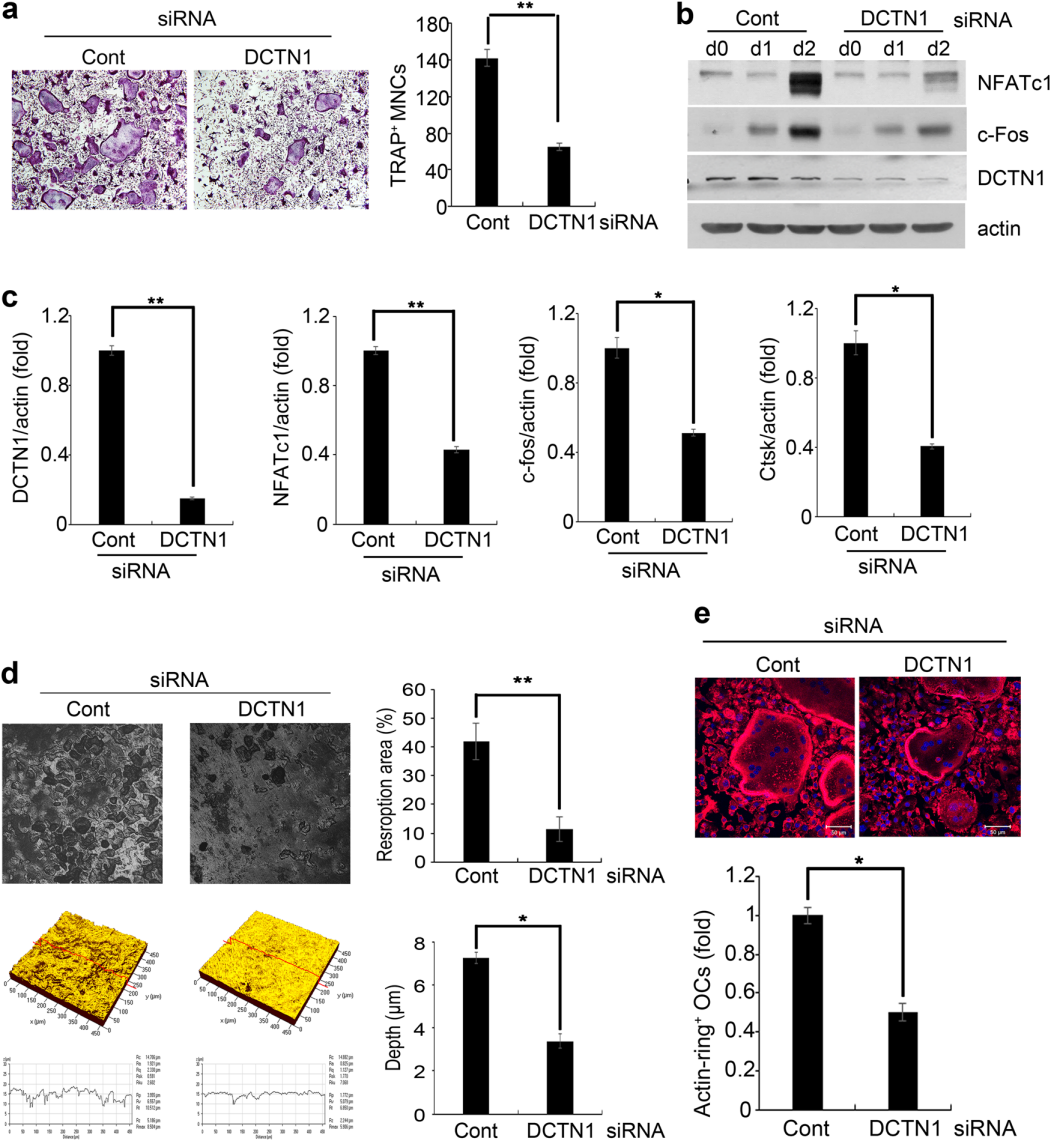
DCTN1 knockdown decreases OC differentiation and bone-resorbing activity. **a** BMMs transfected with control or DCTN1 siRNA were cultured in ODM for 4 days and stained for TRAP. TRAP-positive multinucleated cells were counted. **b** BMMs transfected with control or DCTN1 siRNA were cultured in ODM for the indicated number of days. NFATc1, c-Fos, and DCTN1 protein levels were determined by western blotting. **c** BMMs transfected with control or DCTN1 siRNA were cultured with ODM for 48 h. DCTN1, NFATc1, c-Fos, and Ctsk mRNA levels were analyzed by real-time PCR. **d** BMMs transfected with control or DCTN1 siRNA were cultured on dentin slices in ODM for 9 days. The black areas indicate the resorbed surfaces on dentin slices. The resorption area and pit depth were analyzed by confocal microscopy. **e** BMMs transfected with control or DCTN1 siRNA were cultured in ODM for 4 days. The cells were stained with Alexa Fluor 633-conjugated phalloidin. *p < 0.05, **p < 0.005 compared with controls.

We next investigated whether DCTN1 is involved in OC differentiation using siRNA. BMMs were transfected with DCTN1 or control siRNA. Compared with control siRNA transfection, DCTN1 siRNA transfection greatly decreased the formation of OCs (TRAP^+^ MNCs) (Fig. 2a). An efficient reduction in DCTN1 expression by siRNA was verified at both the protein and mRNA levels (Fig. 2b, c). The RANKL-mediated induction of NFATc1 and c-Fos, transcription factors crucial for OC differentiation, was potently suppressed at both the mRNA and protein levels by DCTN1 silencing (Fig. 2b, c). In line with the reduced osteoclastogenesis, the mRNA expression of cathepsin K (Ctsk), an enzyme important for bone resorption, was decreased by DCTN1 siRNA (Fig. 2c).

Since reduced OC differentiation results in fewer OCs capable of resorbing bone, we performed resorption assays on dentin slices. Consistent with the decrease in OC differentiation, DCTN1 knockdown significantly reduced the resorption area (Fig. 2d). The depth of the resorption pits was also decreased by DCTN1 silencing (Fig. 2d). The reorganization of the actin cytoskeleton into an actin-ring structure is important for bone-resorbing function^1,20^. Therefore, we further investigated the effect of DCTN1 silencing on actin ring formation, which was less evident in DCTN1-silenced OCs than in controls (Fig. 2e, top). The number of actin-ring-positive OCs was also reduced in DCTN1-silenced cells compared with control siRNA-transfected cells (Fig. 2e, bottom). Reductions in pit depth and actin-ring density indicate lower resorbing activity of OCs. The cell migration of precursor cells and fusion of migrated cells are also crucial steps of osteoclast differentiation and are regulated by cytoskeletal rearrangement. The migration of both BMMs and pOCs (Supplementary Fig. 1) and fusion of pOCs into mature multinucleated OCs (Supplementary Fig. 2) were also inhibited by siDCTN1. Taken together, these results suggest that DCTN1 is necessary for the proper function and differentiation of OCs.

### DCTN1 mediates the RANKL activation of Cdc42

**Fig. 3:**
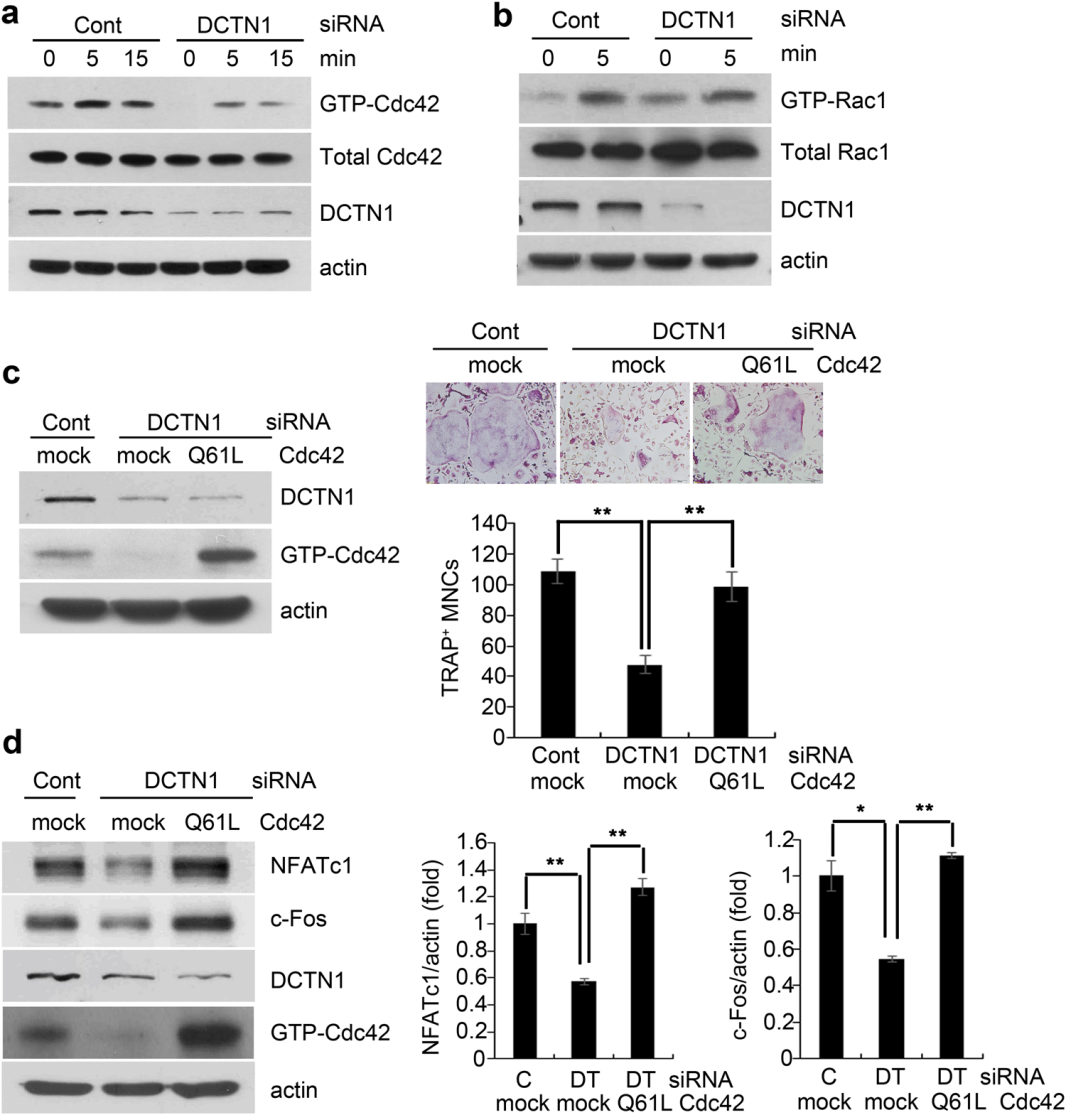
Cdc42 activity is regulated by DCTN1. **a**, **b** BMMs were transfected with control or DCTN1 siRNA. After serum starvation for 4 h, cells were treated with RANKL (400 ng/ml) for the indicated time. The levels of GTP-Cdc42 and GTP-Rac1 were determined by western blotting after active GTP-bound forms of Cdc42 and Rac1 were pulled down. **c** BMMs transfected with control or DCTN1 siRNA were infected with a retrovirus containing Cdc42 Q61L or control DNA. The cells were then cultured in ODM for 4 days and stained for TRAP. TRAP-positive multinucleated cells were counted. DCTN1 and GTP-Cdc42 protein levels were determined as above. **d** BMMs transfected with control or DCTN1 siRNA were infected as in *C* and then cultured in ODM for 48 h. NFATc1 and c-Fos protein levels were determined by western blotting (left panel). NFATc1 and c-Fos mRNA levels were analyzed by real-time PCR (right panel). *p < 0.05, **p < 0.005 compared with controls. Mock, control pMX vector; Q61L, pMX vector containing constitutively active Cdc42.

The Rho family GTPase Cdc42 has been implicated in OC differentiation and function^21^, while the cooperation of Cdc42 with the dynein/dynactin complex in cytoskeleton rearrangement has been demonstrated in fibroblasts^10^. Therefore, we postulated that Cdc42 may be involved in the mechanism by which DCTN1 regulates OC differentiation. To test this possibility, we compared Cdc42 activity in BMMs transfected with DCTN1 siRNA and those transfected with control siRNA after stimulation with RANKL. The active GTP-bound form of Cdc42 was pulled down with beads conjugated to the p21-binding domain, and the amount of precipitated Cdc42 was analyzed by western blotting. As shown in Fig. 3a, DCTN1 knockdown significantly reduced the activation of Cdc42 by RANKL. In contrast, the activation of Rac1, another Rho family GTPase reported to regulate OC differentiation^22,23^, was not affected by DCTN1 knockdown (Fig. 3b).

We next investigated whether Cdc42 activation is essential for the regulation of DCTN1-dependent OC differentiation by evaluating the effect of the overexpression of constitutively active Cdc42 (Q61L) on osteoclastogenesis in DCTN1-silenced BMMs. Efficient overexpression of active Cdc42 was achieved by a retroviral transduction system (Fig. 3c, d, left). The results showed that the impairment of OC differentiation induced by DCTN1 silencing was restored by the overexpression of Cdc42-Q61L (Fig. 3c, right). Consistent with these findings, the inhibition of the RANKL-mediated induction of NFATc1 and c-Fos by DCTN1 knockdown was also reversed by Cdc42-Q61L overexpression (Fig. 3d, right). These results indicate that DCTN1 regulates OC differentiation by mediating the activation of Cdc42 by RANKL.

### PAK2 is a downstream target of DCTN1/Cdc42 in osteoclastogenesis

**Fig. 4:**
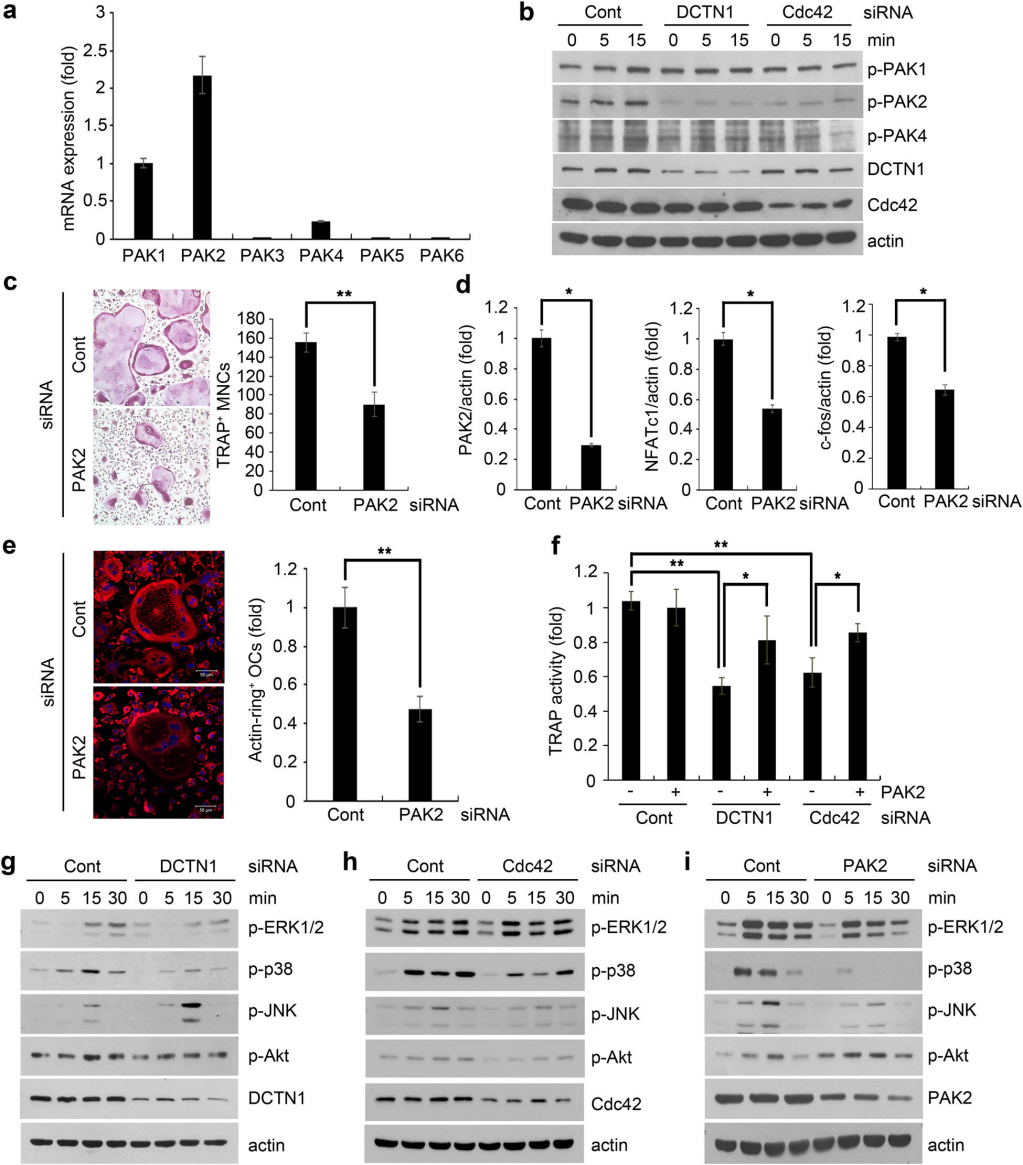
PAK2 is a downstream target of Cdc42 in the DCTN1-mediated regulation of OC differentiation. **a** BMMs were treated with ODM for 48 h. PAK1-6 mRNA levels were analyzed by real-time PCR. **b** BMMs were transfected with control, DCTN1, or Cdc42 siRNA. The cells were treated with RANKL (400 ng/ml) for the indicated time after serum starvation for 6 h. The phosphorylation of PAK1, PAK2, and PAK4 was determined by western blotting. **c**, **d** BMMs were transfected with control or PAK2 siRNA. The cells were cultured in ODM for 4 days and stained for TRAP. TRAP-positive multinucleated cells were counted. PAK2, NFATc1, and c-Fos mRNA levels were analyzed by real-time PCR. **e** BMMs transfected with siRNA were cultured on dentin slices in ODM for 9 days. To visualize the actin-ring structure, the cells were stained with Alexa Fluor 633-conjugated phalloidin. **f** BMMs infected with a retrovirus harboring PAK2 or mock DNA were transfected with control, DCTN1, or Cdc42 siRNA. The cells were cultured in ODM for 5 days, and TRAP activity was measured in cell lysates. **g**, **h**, **i** BMMs were transfected with control, DCTN1, Cdc42, or PAK2 siRNA. The cells were treated with RANKL (400 ng/ml) for the indicated time after serum starvation for 6 h. The phosphorylation of ERK1/2, p38, JNK, and Akt was determined by western blotting. *p < 0.05, **p < 0.005 compared with controls.

We next investigated the potential involvement of PAK family kinases, which act downstream of small GTPases in diverse cellular processes^11^. The six members of the PAK family are classified into two groups, group I (PAK1, PAK2, and PAK3) and group II (PAK4, PAK5, and PAK6), based on structure and function^24^. Since DCTN1 knockdown led to a decrease in RANKL-induced Cdc42 activation, we hypothesized that PAKs play a role in the DCTN1-mediated regulation of osteoclastogenesis. To test this hypothesis, we first examined the expression of the six PAK family members. Quantitative PCR analyses showed that PAK1 and PAK2 mRNAs were dominantly expressed during OC differentiation, while PAK4 mRNA was expressed at a much lower level (Fig. 4a). No expression of PAK3, PAK5, or PAK6 mRNAs was detected. We next evaluated the activation of PAK1, PAK2, and PAK4 by western blotting with antibodies specific for phosphorylated PAKs. RANKL induced the activation of PAK1 and PAK2 (Fig. 4b), and DCTN1 or Cdc42 knockdown by siRNA blocked the activation of PAK2 by RANKL. However, the phosphorylation of PAK1 was not affected by DCTN1 knockdown.

Since PAK2 activation was inhibited by both DCTN1 siRNA and Cdc42 siRNA, we next explored the role of PAK2 in OC differentiation. BMMs were transfected with PAK2 siRNA and cultured with RANKL. PAK2 silencing suppressed the formation of TRAP^+^ MNCs and the induction of c-Fos and NFATc1 (Fig. 4c, d). PAK2 knockdown also decreased the number of mature OCs with an intact actin-ring structure and the resorption area formed on dentin slices (Fig. 4e and Supplementary Fig. 3). Moreover, the overexpression of PAK2 restored the decrease in TRAP activity by DCTN1 or Cdc42 knockdown (Fig. 4f and Supplementary Fig. 4). These results indicate that PAK2 functions downstream of Cdc42 in the DCTN1-mediated control of osteoclastogenesis.

The Cdc42/PAK pathway has been linked to the activation of MAPK and Akt^25,26^, the signaling molecules activated by RANKL for OC differentiation. Therefore, we next examined the effects of DCTN1 and PAK2 silencing on the activation of MAPKs and Akt by RANKL in BMMs. The activation of ERK, p38, and JNK was induced by RANKL, while DCTN1 knockdown attenuated the RANKL-induced activation of ERK1/2 and p38 (Fig. 4g).

The stimulation of p38 phosphorylation by RANKL was also inhibited by both Cdc42 and PAK2 knockdown, while ERK phosphorylation was inhibited by PAK2 knockdown but not by Cdc42 knockdown (Fig. 4h, i). Intriguingly, DCTN1 knockdown enhanced JNK1 activation, whereas Cdc42 and PAK2 knockdown suppressed the activation of JNK1 (Fig. 4g–i). In contrast, DCTN1 and Cdc42 knockdown mitigated Akt activation, whereas PAK2 knockdown augmented Akt activation (Fig. 4g, h). These results suggest that p38 MAPK may play a key role in the DCTN1-Cdc42-PAK2 signaling axis to regulate NFATc1 induction and osteoclastogenesis by RANKL.

### DCTN1 regulates apoptosis during osteoclastogenesis

**Fig. 5:**
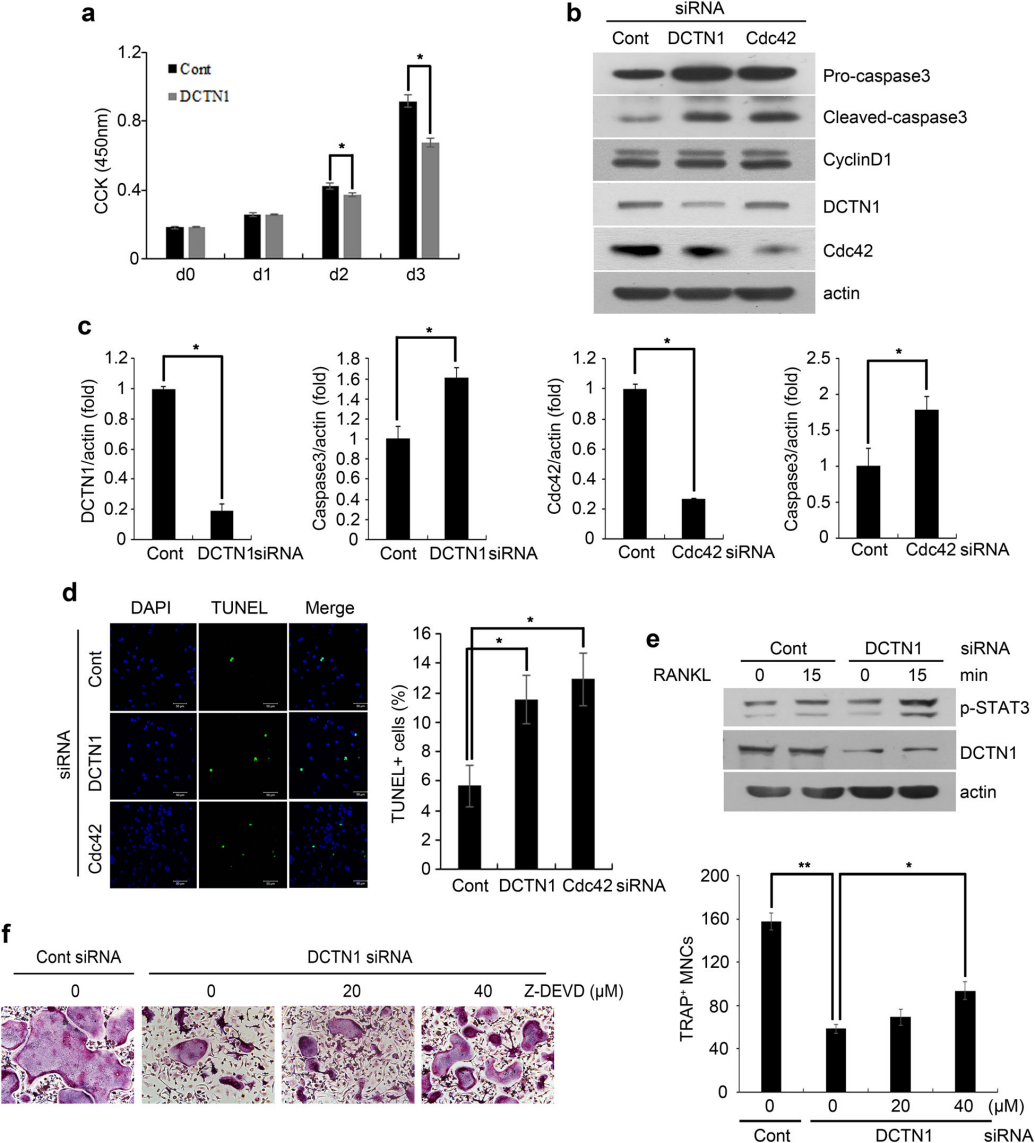
DCTN1 deficiency induces the apoptosis of OC precursors. **a** BMMs transfected with control or DCTN1 siRNA were cultured in ODM for the indicated number of days. A cell proliferation assay was performed with a CCK kit. **b**, **c** BMMs were transfected with control, DCTN1, or Cdc42 siRNA and cultured in ODM for 2 days. The protein and mRNA levels of caspase-3, cyclin D1, DCTN1, and Cdc42 were detected by western blotting and real-time PCR, respectively. **d** BMMs transfected with control, DCTN1, or Cdc42 siRNA were cultured in ODM for 2 days. The cells were subjected to the TUNEL assay, and TUNEL-positive cells were counted. **e** BMMs were transfected with control or DCTN1 siRNA. After serum starvation for 6 h, the cells were treated with RANKL (400 ng/ml) for 15 min. The phosphorylation of STAT3 was determined by western blotting. **f** BMMs transfected with siRNA were cultured in ODM for 4 days with Z-DEVD-FMD or vehicle during the first 48 h. TRAP-positive multinucleated cells were counted. *p < 0.05, **p < 0.005 compared with controls.

Dynactin and dynein have been reported to have roles in cell proliferation and apoptosis as well as intracellular transport^27,28^. As a decrease in proliferation or an increase in apoptosis during osteoclastogenesis might lead to reduced OC formation, we next examined whether DCTN1 knockdown affects the number of OC precursors. We observed a difference in cell number between the control siRNA- and DCTN1 siRNA-treated groups beginning 2 days after RANKL treatment, and this deviation was greater on day 3 (Fig. 5a). However, the expression level of cyclin D1, a proliferation marker, was not altered by DCTN1 or Cdc42 knockdown (Fig. 5b). We also found that DCTN1 or Cdc42 silencing promoted the cleavage of caspase-3 and expression of pro-caspase-3 during osteoclastogenesis (Fig. 5b). In addition, the mRNA level of caspase-3 was upregulated by silencing either DCTN1 or Cdc42 (Fig. 5c), suggesting that caspase-3 was regulated at the transcriptional level. Consistent with these results, the number of TUNEL-positive apoptotic cells was increased by DCTN1 or Cdc42 knockdown (Fig. 5d).

A recent study showed that phospho-STAT3 binds to the caspase-3 promoter and stimulates caspase-3 transcription in muscle cells^29^. Therefore, we investigated whether DCTN1 knockdown affects STAT3 activation. The phosphorylation of STAT3 induced by RANKL was greater when DCTN1 was silenced (Fig. 5e). To gain further evidence for the contribution of apoptosis to the anti-osteoclastogenic effect of DCTN1 silencing, we next cultured BMMs transfected with DCTN1 siRNA in the presence or absence of Z-DEVD-FMK, a caspase-3 inhibitor. Z-DEVD-FMK treatment partially restored OC generation from DCTN1-silenced BMMs (Fig. 5f). Collectively, these results indicate that the DCTN1-mediated regulation of osteoclastogenesis is in part achieved by suppressing apoptosis via STAT3 inhibition and caspase-3 reduction.

### DCTN1 knockdown reduces osteoclastogenesis and bone resorption in vivo

**Fig. 6:**
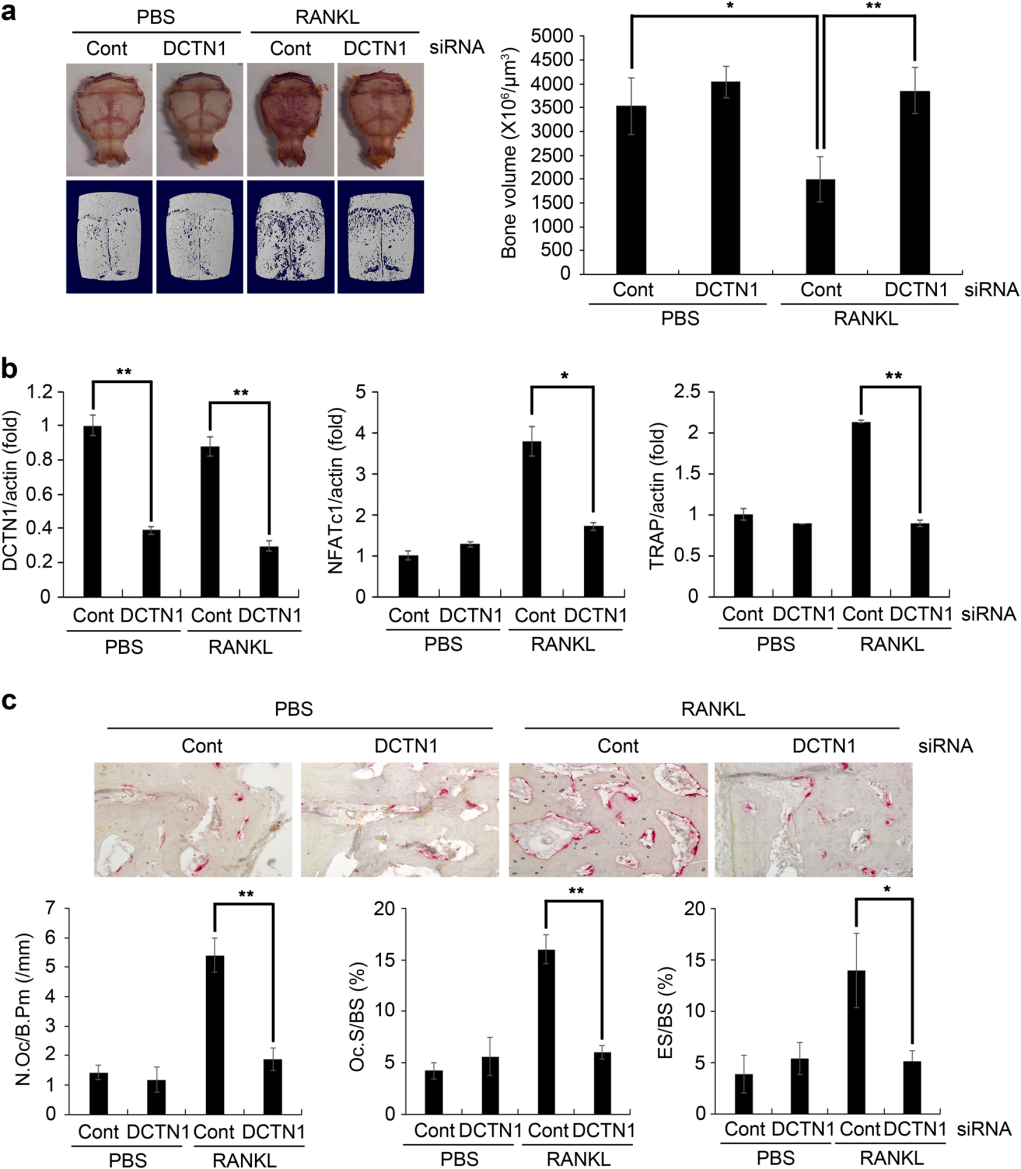
DCTN1 silencing decreases osteoclastogenesis in vivo. Control or DCTN1 siRNA mixed with transfection reagents was injected into the calvariae of 5-week-old mice, and then a collagen sheet soaked with PBS or RANKL was implanted. On day 7, the mice were sacrificed. **a** Mouse calvariae were stained for TRAP (upper panel). The calvariae were also subjected to μCT analysis, and 3D images are shown (lower panel). Quantitative values of calvarial bone volume are shown in a histogram (right panel). **b** DCTN1, NFATc1, and TRAP mRNA levels in calvarial tissue were analyzed by real-time PCR. **c** Sections of calvariae were stained for TRAP activity. Representative images of stained sections are shown. Quantitative analysis of the sections included the following parameters: OC number/bone perimeter (N.Oc/B.Pm); OC surface/bone surface (Oc.S/BS); and eroded surface/bone surface (ES/BS). *n* = 5 per group. *p < 0.05, **p < 0.005 compared with PBS control.

We next examined the effect of DCTN1 siRNA on osteoclastogenesis and bone resorption in vivo. Control or DCTN1 siRNA oligonucleotides mixed with transfection reagents were injected on the calvariae of mice that received either a PBS- or RANKL-soaked collagen implant. At 7 days after implantation, whole calvariae were stained for TRAP activity and subjected to μCT analysis. In the mice with RANKL-treated implants, DCTN1 siRNA injection reduced the intensity of TRAP staining in the calvariae (Fig. 6a). Furthermore, μCT analyses clearly showed that RANKL treatment caused a prominent reduction in bone volume, while RANKL-induced bone loss was blocked in mice injected with DCTN1 siRNA (Fig. 6a). We confirmed efficient suppression of DCTN1 mRNA in calvarial tissue in the DCTN1 siRNA-injected group (Fig. 6b). The mRNA levels of NFATc1 and TRAP were also decreased by DCTN1 knockdown in vivo. For histological analyses, decalcified mouse calvarial sections were stained for TRAP activity. The control siRNA-injected group showed an increased TRAP-positive area induced by RANKL. In the DCTN1 siRNA-injected groups, little difference in TRAP-positive area was observed between the PBS- and RANKL-treated groups (Fig. 6c, upper panel). Quantitative analyses revealed that compared with control siRNA, DCTN1 siRNA decreased the OC number per bone perimeter (N.Oc/B.Pm), the OC surface per bone surface (Oc.S/BS) and the eroded surface per bone surface (ES/BS) in the RANKL-treated group (Fig. 6c, lower panel).

### Overexpression of DCTN1 interferes with osteoclastogenesis

**Fig. 7:**
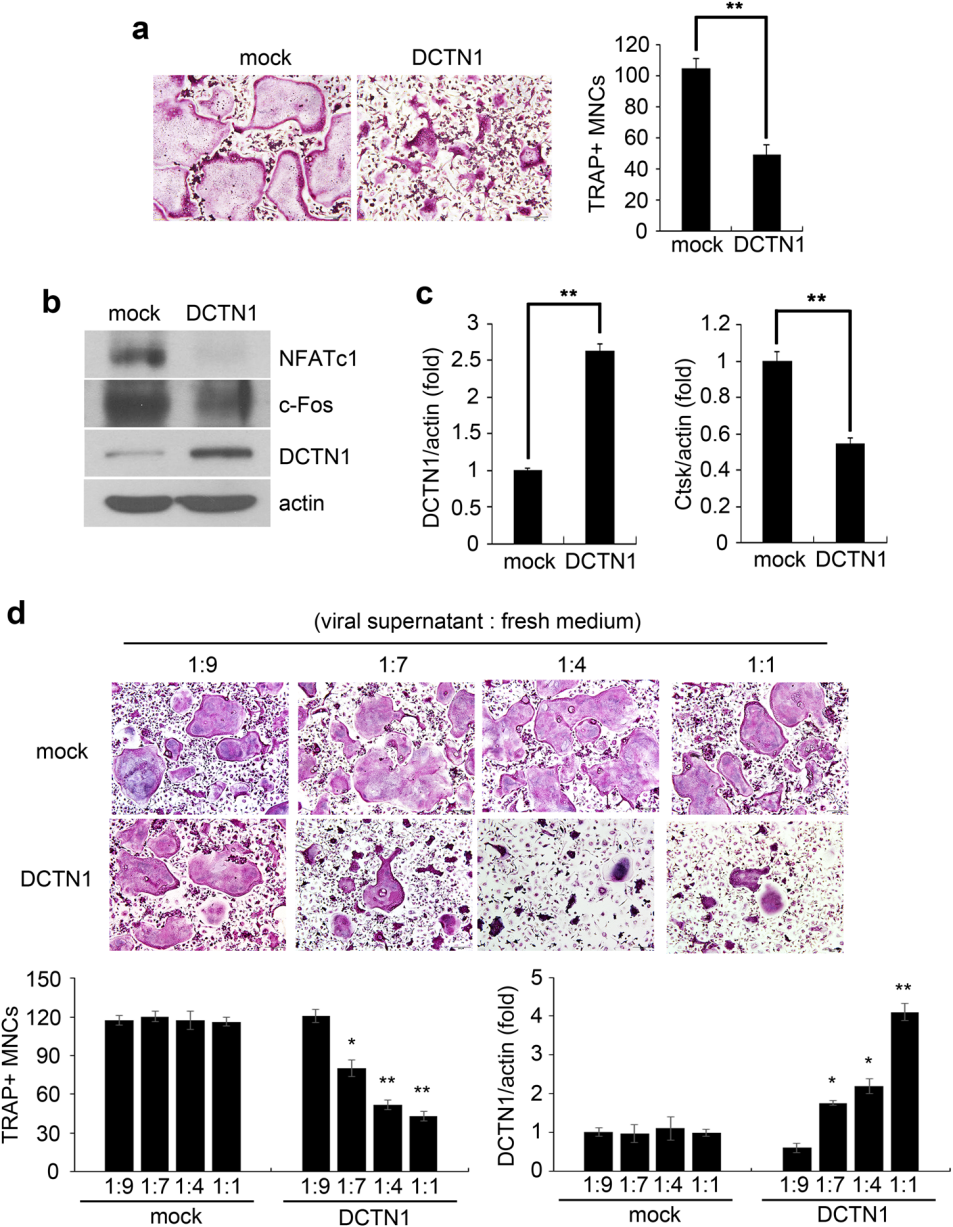
Overexpression of DCTN1 interferes with OC differentiation. **a** BMMs infected with a retrovirus harboring DCTN1 or mock DNA were cultured in ODM for 4 days and stained for TRAP. TRAP-positive multinucleated cells were counted. **b**, **c** BMMs transduced as described in *A* were cultured in ODM for 48 h. NFATc1 and c-Fos protein levels were determined by western blotting. DCTN1 and Ctsk mRNA levels were analyzed by real-time PCR. **d** BMMs were infected with retroviral supernatant diluted at the indicated ratio (retroviral supernatant:fresh medium). Cells were further cultured in ODM for 4 days and stained for TRAP. TRAP-positive multinucleated cells were counted. DCTN1 mRNA levels were analyzed by real-time PCR. *p < 0.05, **p < 0.005 compared with controls. Mock, pMX vector; DCTN1, pMX-DCTN1 vector.

As DCTN1 knockdown led to a reduction in osteoclastogenesis, we speculated that the overexpression of DCTN1 may exhibit a promoting effect on OC formation. Previous studies have reported that the excessive expression of some subunits of the dynactin complex can impair dynactin function^7^. Therefore, DCTN1 overexpression could have either stimulating or suppressing effects on osteoclastogenesis. To test the effects, we infected BMMs with control or DCTN1-expressing retroviruses and cultured the cells in OC differentiation medium. The overexpression of DCTN1 led to significant inhibition of OC formation (Fig. 7a). The overexpression of DCTN1 was verified at both the protein and mRNA levels (Fig. 7b, c). The protein levels of both NFATc1 and c-Fos were also reduced by DCTN1 overexpression (Fig. 7b). Consistent with these results, DCTN1 overexpression decreased the gene expression of Ctsk (Fig. 7c). Based on these results, we hypothesized that quantitative balancing of DCTN1 levels is essential for normal OC differentiation. To address this notion, we examined whether the degree of DCTN1 overexpression correlates with the extent of its anti-osteoclastogenic effect. To this end, culture supernatant containing the DCTN1 retrovirus was added to BMMs at different dilutions to achieve various degrees of DCTN1 overexpression. As shown in Fig. 7d, OC differentiation was inhibited in a manner dependent on the degree of DCTN1 overexpression. Consistent with reduced osteoclastogenesis, RANKL-induced p38 activation was attenuated by DCTN1 overexpression, despite the elevated activation of Cdc42 and PAK2 (Supplementary Fig. 5). Cell apoptosis and cleaved caspase-3 levels were not affected by DCTN1 overexpression (Supplementary Fig. 6). These results suggest that precise control of DCTN1 levels is important for efficient osteoclastogenesis and that p38 may play key roles in the regulation of osteoclastogenesis by DCTN1.

## Discussion

Several motor proteins have been implicated in the regulation of bone metabolism. For example, the motor protein Kif3a has been shown to regulate bone formation by modulating hedgehog, Wnt, and calcium signaling in osteoblasts^30^. In addition, the motor proteins myosin IXB and X have been reported to have a critical role in podosome patterning and the resorption capacity of OCs^31,32^. In this study, we identified a novel link between DCTN1, a crucial regulator of the dynein motor, and OC differentiation.

Our study indicates that DCTN1 plays a role in the regulation of OC differentiation by participating in RANKL signaling-mediated Cdc42/PAK2 activation. Our results showing the involvement of Cdc42 in osteoclastogenesis are consistent with a previous study in which Cdc42-deficient cells exhibited impaired M-CSF- or RANKL-mediated signaling events^21^. The cooperation of Cdc42 and the dynein/dynactin complex has been demonstrated to be involved in microtubule-organizing center reorientation induced by lysophosphatidic acid^10^. However, it has been unclear whether DCTN1 is involved in the activation of Cdc42. In the present study, we revealed that DCTN1 exerts its effects on OC differentiation by activating Cdc42 in OCs (Fig. 3a). The overexpression of Cdc42-Q61L in DCTN1-silenced BMMs restored the formation of OCs and induction of NFATc1 and c-Fos by RANKL (Fig. 3). Activated Cdc42 regulates diverse cellular events by interacting with its effector molecules, such as PAKs^33,34^. PAKs have been shown to mediate multiple cellular processes, including MAPK signaling, cell survival, and cytoskeleton reorganization^11,35^. However, the potential role of PAKs in bone metabolism has not been explored. In our study, PAK2 silencing led to the disruption in RANKL-stimulated MAPK signaling cascades and a decrease in OC generation (Fig. 4). To the best of our knowledge, this is the first report to reveal the involvement of PAK2 in osteoclastogenesis.

A previous study showed that the G59S mutation in DCTN1 disrupts dynactin complex function and induces neuron death^36^. Our experiments revealed that DCTN1 deficiency enhanced the death of OC precursors (Fig. 5). The role of STAT3 in cell death was suggested by a recent study that showed that STAT3 activation directly stimulated caspase-3 transcription^29^. In our study, DCTN1 knockdown increased STAT3 phosphorylation and caspase-3 levels (Fig. 5). Activated STATs translocate to the nucleus for target gene transcription, and the transport of STAT5B to the nucleus in growth hormone-stimulated hepatocytes has been shown to be a dynein-regulated microtubule-dependent process^37^. Whether an analogous mechanism functions in the nuclear translocation of STAT3 remains to be elucidated. However, as DCTN1 knockdown leads to the malfunction of dynein, the effect of DCTN1 silencing on STAT3 is unlikely to be associated with the role of dynein in the nuclear transport of STATs.

Although ERK1/2 and p38 were downregulated in DCTN1-silenced BMMs, JNK phosphorylation was increased (Fig. 4g). Enhanced JNK activation has been shown to increase the cell death of OC precursors and thus decrease RANKL-induced osteoclastogenesis in RelA^−/−^ mice^38^. As we found that the absence of DCTN1 accelerated OC precursor apoptosis upon RANKL treatment (Fig. 5), we tested whether elevated JNK activation reduces OC differentiation. However, JNK suppression by SP600125 did not prevent apoptosis or the inhibition of osteoclastogenesis by DCTN1 silencing (data not shown), suggesting that the apoptosis of OCs induced by DCTN1 knockdown was mainly due to STAT3-mediated caspase-3 induction and not increased JNK activity. It is possible that the upregulation of JNK activation is a part of the compensatory mechanism of ERK and p38 MAPK cascade impairment.

Several subunits of the dynactin complex have been shown to prevent dynactin function when they are excessively expressed. The overexpression of DCTN2, which disrupts dynactin structure and impairs the interaction between dynein and dynactin, has been used as a strategy to block dynein function^39^. The overexpression of other subunits, such as Arp11 and p62, also prevents the binding of dynactin with its cellular targets^7^, suggesting that a balanced level of each subunit of the dynactin complex is important for proper functioning of dynactin. In our study, excessive DCTN1 expression inhibited OC differentiation by RANKL (Fig. 7a). Furthermore, a slight alteration in DCTN1 expression was sufficient to block OC generation (Fig. 7d). In addition, a previous study showed that DCTN2 overexpression leads to suppressed OC formation^40^. These results suggest that maintaining dynactin subunits at proper levels is critical for normal osteoclastogenesis.

In addition to dynein, dynactin subunits have diverse binding partners in the cytoplasm and nucleus^7,41,42^. For example, DCTN2 interacts with calmodulin and macrophage-enriched myristoylated alanine-rich C kinase substrate (MacMARCKS) in macrophages^43^. In MCF-7 cells, DCTN1 has been shown to interact with estrogen receptor α (ERα)^44^. Estrogen induces the recruitment of DCTN1 at the promoter region of estrogen-responsive genes, and the transcriptional activity of ERα is significantly elevated by DCTN1 overexpression^44^. In addition, small amounts of DCTN1 are present in the nucleus, even in the presence of microtubule depolymerization reagents^44^. These results indicate that DCTN1 is involved not only in cargo transportation but also in gene transcription by binding to nuclear partners. Similarly, the dynein light chain is also found in the nucleus and has been shown to bind ERα, promoting the transcription of ERα target genes^45^, and β-tubulin in the nucleus has been reported to act as a transcriptional cofactor of Notch^46^. Estrogen protects against osteoporosis by attenuating bone resorption, and ERα deletion in OC precursors abrogates this effect via the regulation of Fas ligand expression^47^. Based on these reports, we speculate that the enhancement of DCTN1 levels in the nucleus by overexpression might inhibit OC differentiation by modulating the transcription of ERα target genes or other anti-osteoclastogenic genes without impairing the transport function of the dynactin complex.

The underlying mechanism of RANKL-RANK signaling transduction-induced DCTN1 activation is still unclear and should be examined in future studies. DCTN1 has been shown to be phosphorylated at serine residues^48^. In addition, DCTN1 has been suggested to be a direct target of Akt in the mediation of Akt localization to microtubules^17^. This DCTN1-mediated association of Akt with microtubules enhances Akt activation in muscle cells, suggesting a cross-potentiating relationship between Akt and DCTN1 through microtubule binding^17^. In RANKL signaling, Akt has been shown to be activated via the TRAF6-PI3K-PDK pathway^3^. Therefore, it is possible that the RANKL-induced activation of Akt via PI3K leads to the phosphorylation of DCTN1, which in turn facilitates microtubule association and subsequent further activation of Akt. Another question involves the molecular mechanism by which DCTN1 mediates Cdc42 activation in response to RANKL. Whether DCTN1 functions in recruiting a guanine nucleotide exchange factor (GEF) or suppressing a GTPase-activating protein for Cdc42 should be examined. For example, Vav3, a Rho family GEF, has been shown to be crucial for bone resorption^49^. Therefore, DCTN1 may participate in Cdc42 activation by mediating the recruitment of a GEF to Cdc42 during OC differentiation.

**Fig. 8:**
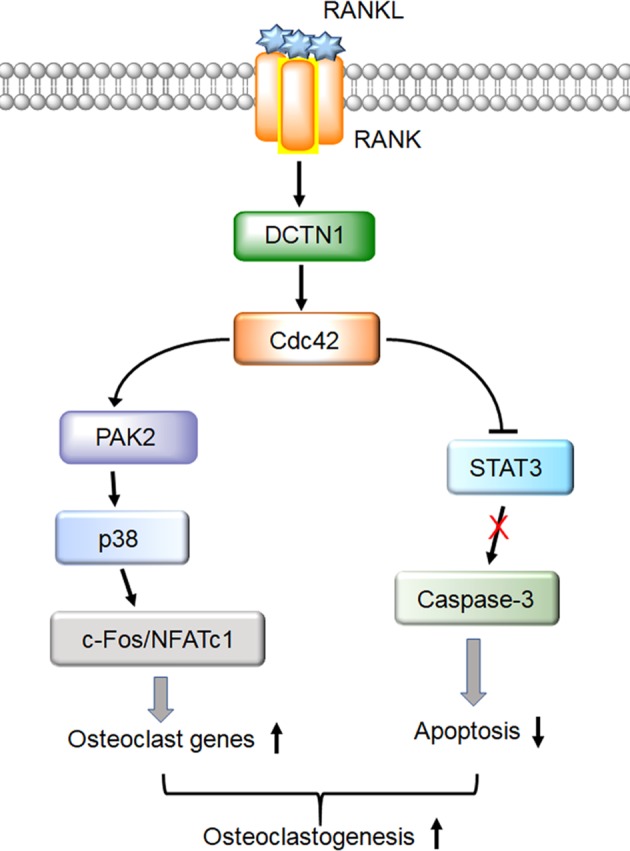
Schematic model of the proposed mechanism by which DCTN1 regulates osteoclastogenesis. The RANKL-RANK interaction promotes DCTN1-mediated Cdc42 activation. Activated Cdc42 enhances PAK2 phosphorylation, which evokes the transcription of both NFATc1 and c-Fos through the p38 MAPK pathway. DCTN1 also contributes to the survival of OC precursors by suppressing STAT3 activation. A balanced level of DCTN1 is critical for OC differentiation, as both deficiencies in and the overexpression of DCTN1 interfere with osteoclastogenesis.

In conclusion, we unveiled the crucial role of DCTN1 in OC differentiation (Fig. 8). DCTN1 mediates RANKL-RANK signaling-mediated Cdc42 activation. Activated Cdc42 then stimulates PAK2, which enhances the activity of p38 MAPK. The subsequent activation and induction of c-Fos and NFATc1 lead to the expression of OC genes. DCTN1 is also involved in the repression of caspase-3, inhibiting the apoptosis of OC precursors. The combined effects of DCTN1 on OC gene expression and cell apoptosis contribute to effective osteoclastogenesis.

## Supplementary information

Supplementary Information
