# Supplementary material for: The dynactin subunit DCTN1 controls osteoclastogenesis via the Cdc42/PAK2 pathway
Source: Exp Mol Med. 2020 Mar 24;52(3):514–28. doi: 10.1038/s12276-020-0406-0 (PMC7156411; doi:10.1038/s12276-020-0406-0)
Supplement: Supplementary file 1 — Supplementary Information [file 12276_2020_406_MOESM1_ESM.docx]

**SUPPLEMENTARY INFORMATION**

**SUPPLEMENTARY MATERIALS AND METHODS**

**Migration assay**

1×10^5^ BMMs or pOCs in 200 μl of serum-free α-MEM were seed in the upper well of a Boyden chamber with polycarbonate filters containing 8-μm pore membranes (Corning Costar, Cambridge, MA). The lower well was loaded with 600 μl of serum-free α-MEM containing 100 ng/ml of RANKL. After 8 hours for BMMs and 12 hours for pOCs of incubation, cells attached on the lower surface of the membrane were fixed with 100% methanol for 10 min and then stained with crystal violet solution (Sigma, St. Louis, MO, USA). Migrated cells were quantified by using an image analysis system (Image J, National Institutes of Health, Bethesda, MD).

**Osteoclasts fusion assay**

Osteoclasts fusion assay with LPC block was assessed as previously reported^1^. Briefly, a 10 mg/ml stock solution of lauroyl-LPC (1-lauroyl-2-hydroxysn-glycero-3-phosphocholine) (Avanti Polar Lipids) was freshly prepared in water. To block osteoclast fusion, BMMs were cultured with ODM containing of 170 μM LPC for 72 hours. Then, cells were further cultured with ODM contacting of 340 μM LPC for 16 hours. After then, LPC was removed by five washes with LPC-free culture medium and cells were incubated with LPC-free ODM for 1.5 hours. After TRAP staining, TRAP-positive cells with three more nuclei were quantified by using the Image J software.

**Flow cytometry**

Cell apoptosis was assayed with FITC Annexin V Apoptosis Detection Kit (BD Pharmingen, CA, USA) according to manufacturer’s instruction. In briefly, BMM cells (5×10^5^) were cultured in a 60-mm petri dish. After appropriate treatments, cells were harvested and washed with PBS, followed by suspension in 1× binding buffer. Cells were stained with annexin V-FITC and PI mixture for 15 min at room temperature in the dark. Apoptotic cells were identified using a BD FACSAria III flow cytometer (BD biosciences, CA, USA). All flow cytometry data were analyzed with FlowJo software (Ashland, OR, USA).

1 Verma, S. K., Leikina, E., Melikov, K. & Chernomordik, L. V. Late stages of the synchronized macrophage fusion in osteoclast formation depend on dynamin. *The Biochemical journal* **464**, 293-300, doi:10.1042/bj20141233 (2014).

**SUPPLEMENTARY TABLE**

**Supplementary Table 1.** The sequences of primers for real-time PCR.

| **Gene** |  | **Sequence** | **GenBank accession #** |
| --- | --- | --- | --- |
|  |  | 5’- -3’ |  |
| DCTN1 | Sense | TTGACGTGGGTGGTAGCTGT | NM_007835.2 |
|  | Antisense | TCTGCGTCATACTCGCCTTC |  |
| DCTN2 | Sense | CAGATGCTGCAATCAACCTT | NM_001190454.1 |
|  | Antisense | CAACTTTGGCAGCTTGAGAG |  |
| DCTN2 | Sense | CAGTTCATCCTCTCCCAGGT | NM_016890.4 |
|  | Antisense | ATACCCTTCCAGGAGAGCCT |  |
| DCTN4 | Sense | CACACACAACGGATGAACAA | NM_026302.3 |
|  | Antisense | AGGGAAAGTCCAGCAAGTGT |  |
| DCTN5 | Sense | ACGTTGGGAAGAACTGTGTG | NM_021608.3 |
|  | Antisense | AATCATCAGCTCCTGTGTGC |  |
| DCTN6 | Sense | TCAACACCTTTGAAGCCATC | NM_001293757.1 |
|  | Antisense | GCTGCCTTTCATCGTCTTCT |  |
| Arp1 | Sense | ACTTGATTGGCGAGGAGAGT | NM_016860.1 |
|  | Antisense | TCACTCAGTAGCCTGTCTCCA |  |
| Arp11 | Sense | TGTGGTTATCGAGTCGGTGT | AF190797.1 |
|  | Antisense | CCAGGCTTTCCCTATATCCA |  |
| CapZα | Sense | CAGTTCACACCCGTGAAGAT | NM_009797.2 |
|  | Antisense | CTCCCTCCAAGACTTCAAGC |  |
| CapZβ | Sense | ACCCTCCTTTGGAAGATGG | NM_001037761.2 |
|  | Antisense | TGAGGATCACTCCAGCAAAG |  |
| PAK1 | Sense | TGCCGAGAGTGTCTACAAGC | NM_011035.2 |
|  | Antisense | CCATCCAATATGGAGTTCCC |  |
| PAK2 | Sense | AGAAGAAGAACCCTCAGGCA | NM_177326.3 |
|  | Antisense | GCAGCGTCTTCATCATCATC |  |
| PAK3 | Sense  Antisense | ACATTGCGACTGGACAAGAG  AAGAGCCACCAGCCAATAT | NM_001195046.1 |
| PAK4 | Sense  Antisense | GAGAGAGTCCACCACCACCT  GACCATCCCTCGAAGATTTG | NM_027470.3 |
| PAK5 | Sense  Antisense | CAAGGAGTGATTCACAGGGA  GTCCCATAAGGTAGCCTGGA | NM_172858.2 |
| PAK6 | Sense  Antisense | CCTCTTCAAACCTGGTAGCC  ATTGCTGGTGCTGATCTGAG | NM_001033254.3 |
| NFATc1 | Sense  Antisense | CCAGTATACCAGCTCTGCCA  GTGGGAAGTCAGAAGTGGGT | NM_016791.4 |
| c-fos | Sense  Antisense | ACTTCTTGTTTCCGGC  AGCTTCAGGGTAGGTG | NM_010234 |
| Ctsk | Sense  Antisense | ATATGTGGGCCACCATGAAAGTT  TCGTTCCCCACAGGAATCTCT | NM_007802.4 |
| Cdc42 | Sense  Antisense | CAACAAACAAATTCCCATCGC  TGAGGATGGAGAGACCACTG | NM_009861.3 |
| Caspase-3 | Sense  Antisense | AAGGAGCAGCTTTGTGTGTG  GTCTCAATGCCACAGTCCAG | NM_001284409.1 |
| β-actin | Sense | TCTGGCACCACACCTTCTAC | NM_007393.5 |
|  | Antisense | TACGACCAGAGGCATACAGG |  |

**SUPPLEMENTARY FIGURES**


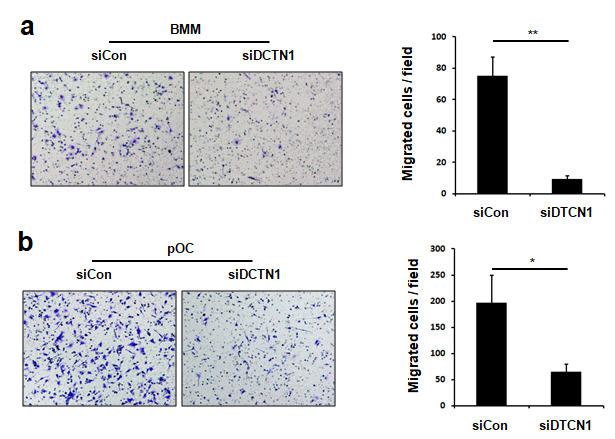


**Supplementary Figure 1** DCTN1 knockdown decreases cell migration. (**a**) Cell migrations of BMMs transfected with control or DCTN1 siRNA were assessed in transwell chambers with RANKL (100 ng/ml) for 8 hours. (**b**) BMMs transfected with control or DCTN1 siRNA were cultured with ODM for 2 days. Then cell migrations of pOCs were assessed in transwell chambers with RANKL (100 ng/ml) for 12 hours. *p < 0.05, **p < 0.005 compared with controls.


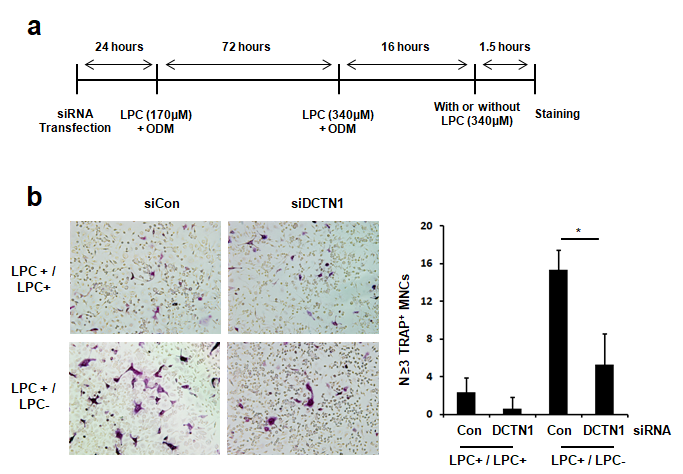


**Supplementary Figure 2** DCTN1 knockdown decreases cell fusion of osteoclasts. (**a**) Experimental scheme of osteoclast fusion assay with LPC. (**b**) BMMs transfected with control or DCTN1 siRNA were cultured with ODM containing LPC (170 μM) for 72 hours and further cultured with ODM containing LPC (340 μM) for 16 hours. Ready-to-fuse osteoclasts were then incubated in ODM with (LPC+ / LPC+) or without (LPC+ / LPC-) LPC (340 μM) for 1.5 hours. Osteoclasts with three or more nuclei (N ≥ 3) were quantified. *p < 0.05 compared with controls.


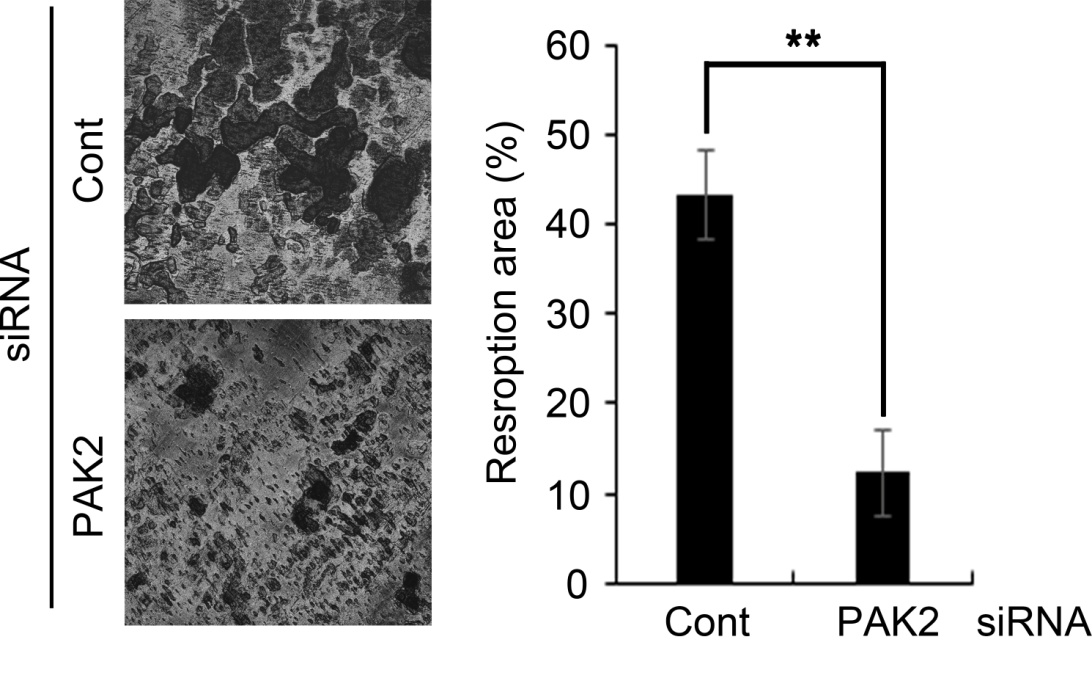


**Supplementary Figure 3** PAK2 regulates bone resorption activity of osteoclasts. BMMs transfected with siRNA were cultured on dentin slices in ODM for 9 days. The resorption area and pit depth were measured. **p < 0.005 compared with controls.


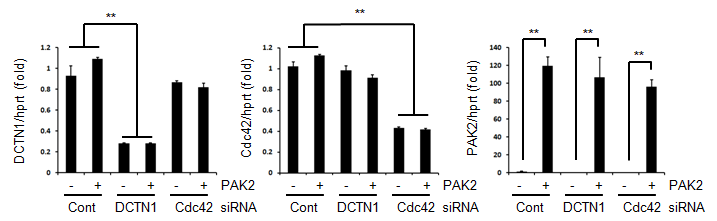


**Supplementary Figure 4** BMMs infected with retrovirus harboring PAK2 or mock DNA were transfected with control-, DCTN1-, or Cdc42-siRNA. mRNA levels of DCTN1, Cdc42, and PAK2 were analyzed by real-time PCR. **p < 0.005 compared with indicated controls.

**
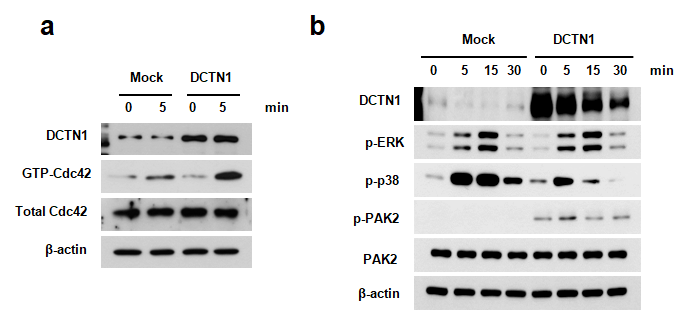
**

**Supplementary Figure 5** Effects of DCTN1 overexpression on the activity of downstream signaling molecules. (**a**) BMMs were infected with retrovirus harboring DCTN1 or mock DNA. After serum starvation for 4 h, cells were treated with RANKL (300 ng/ml) for the indicated times. The levels of GTP-Cdc42 were determined by western blotting after pull-down of active GTP-bound forms of Cdc42. **(b)** BMMs were infected with retrovirus harboring DCTN1 or mock DNA. Then cells were treated with RANKL (300 ng/ml) for the indicated time after serum starvation for 6 h. The phosphorylation of ERK1/2, p38, JNK, and PAK2 was determined by western blotting.


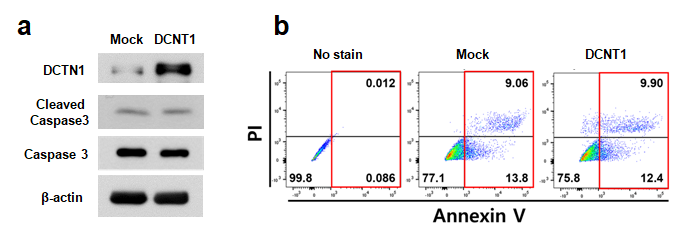


**Supplementary Figure 6** DCTN1 overexpression does not affect cell apoptosis during osteoclastogenesis. (**a, b**) BMMs were infected with retrovirus harboring DCTN1 or mock DNA and cultured with ODM for 2 days. (**a**) Protein levels of DCTN1, cleaved-caspase-3, caspase-3 were determined by western blotting. **(b)** PI uptake and cell surface annexin V were detected by flow cytometry.
